# A Toxin-Antitoxin Module in *Bacillus subtilis* Can Both Mitigate and Amplify Effects of Lethal Stress

**DOI:** 10.1371/journal.pone.0023909

**Published:** 2011-08-29

**Authors:** Xiangli Wu, Xiuhong Wang, Karl Drlica, Xilin Zhao

**Affiliations:** 1 Public Health Research Institute, New Jersey Medical School, University of Medicine and Dentistry of New Jersey, Newark, New Jersey, United States of America; 2 State Key Laboratory for Agrobiotechnology and Department of Microbiology, China Agricultural University, Beijing, China; 3 Department of Biochemistry, Harbin Medical University, Harbin, Heilongjiang Province, China; 4 Department of Microbiology & Molecular Genetics, New Jersey Medical School, University of Medicine and Dentistry of New Jersey, Newark, New Jersey, United States of America; Duke University Medical Center, United States of America

## Abstract

**Background:**

Bacterial type-2 (protein-protein) toxin-antitoxin (TA) modules are two-gene operons that are thought to participate in the response to stress. Previous work with *Escherichia coli* has led to a debate in which some investigators conclude that the modules protect from stress, while others argue that they amplify lethal stress and lead to programmed cell death. To avoid ambiguity arising from the presence of multiple TA modules in *E. coli*, the effect of the sole type-2 toxin-antitoxin module of *Bacillus subtilis* was examined for several types of lethal stress.

**Methodology/Principal Findings:**

Genetic knockout of the toxin gene, *ndoA* (*ydcE*), conferred protection to lethal stressors that included kanamycin, moxifloxacin, hydrogen peroxide, and UV irradiation. However, at low doses of UV irradiation the *ndoA* deficiency increased lethality. Indeed, gradually increasing UV dose with the *ndoA* mutant revealed a crossover response – from the mutant being more sensitive than wild-type cells to being less sensitive. For high temperature and nutrient starvation, the toxin deficiency rendered cells hypersensitive. The *ndoA* deficiency also reduced sporulation frequency, indicating a role for toxin-antitoxin modules in this developmental process. In the case of lethal antimicrobial treatment, deletion of the toxin eliminated a surge in hydrogen peroxide accumulation observed in wild-type cells.

**Conclusions:**

A single toxin-antitoxin module can mediate two opposing effects of stress, one that lowers lethality and another that raises it. Protective effects are thought to arise from toxin-mediated inhibition of translation based on published work. The enhanced, stress-mediated killing probably involves toxin-dependent accumulation of reactive oxygen species, since a deficiency in the NdoA toxin suppressed peroxide accumulation following antimicrobial treatment. The type and perhaps the level of stress appear to be important for determining whether this toxin will have a protective or detrimental effect.

## Introduction

Type-2 (protein-protein) toxin-antitoxin (TA) gene pairs were initially described as “addiction modules” that allow stable maintenance of low-copy-number plasmids (reviewed in [1]). The two protein-encoding genes in each module are co-expressed, with the activity of the stable toxin being inhibited by the labile antitoxin via tight protein-protein interactions [2]. Proteolytic removal of the antitoxin leads to toxin activation [2]. When the TA gene module is lost and cells are unable to maintain continuous antitoxin production, rapid degradation of antitoxin allows residual toxin to kill cells. Thus, plasmid-free daughter cells are eliminated from bacterial populations. TA modules were subsequently found as chromosomal genes in a wide variety of bacteria where they encode factors that may participate in bacterial responses to stressful conditions [2], [3].

One line of work with the *Escherichia coli* stress response suggested that at least one chromosomal TA module (*mazEF*) triggers programmed cell death when activated by stresses such as antimicrobials, heat, UV irradiation, thymine starvation, high concentrations of ppGpp, and hydrogen peroxide [4], [5], [6], [7]. However, other laboratories argue that TA modules contribute to bacterial stasis (persistence), thereby facilitating survival during stress rather than apoptosis [2], [8], [9], [10], [11]. Examples of the latter derive from results with nutrient depletion and ectopic overproduction of a toxin or TA pair [8], [11], [12], [13], [14]. An unequivocal phenotype with a TA knockout would help clarify TA function. Unfortunately, reported phenotypes of TA module knockout mutants are complex and sometimes even controversial. For example, the enhanced killing effects observed by one research group [4], [5], [6], [7] could not be verified by others ([15]; Wang and Zhao, unpublished observation). But more recently Davis *et al* observed that deletion of *mazEF*, particularly when combined with a deletion of *relBE*, protects cells from hydroxylurea-mediated killing [16]. Then Kim and Wood reported that deletion of the *mqsR* toxin gene reduces persistence [17]. No simple rule has emerged. Indeed, even more complexity arises from the observation that deletion of five TA systems (*mazEF, relBE, chpBIK, yoeB/yefM, and yafQ/dinJ*) decreased biofilm formation initially (8 hr) but increased biofilm formation at a later time (24 hr) by decreasing biofilm dispersal [18]. Moreover, a phenotype opposite to that seen when all five TA systems were deleted was observed when one of the five TA systems was deleted [18]. General conclusions about toxin function may have been difficult to draw because *E. coli* contains more than a dozen TA modules that are likely to be partially redundant [3], [19].

One way to unequivocally define a phenotype for TA modules is to examine a model system having one or only a few modules so that all of the TA genes can be readily inactivated. *Bacillus subtilis* offers such a system, since searches for all canonical protein-protein (type-2) TA modules, by several genomic surveys using various algorithms, reveal only a single TA pair [3], [19], [20]. In the unique *B. subtilis* TA module, *ndoA* (*ydcE*) encodes an endoribonuclease that behaves like the MazF toxin of *E. coli* [21]. The antitoxin gene, *ndoAI* (*ydcD*), which is located upstream from *ndoA* in the same two-gene operon, encodes a MazE-like antitoxin protein that blocks NdoA toxicity [21]. Inactivation of the toxin gene can be readily achieved via homologous recombination using an antibiotic cassette flanked with toxin gene sequences [21], [22].

In the present work, we prepared an *ndoA* (toxin) knock-out mutant of *B. subtilis* and measured bacterial survival following administration of several stressors at a variety of doses. The *ndoA* deficiency lowered lethal effects of some antimicrobials, such as kanamycin and moxifloxacin, and environmental stressors, such as hydrogen peroxide. However, for other types of stress, such as high temperature, the *ndoA* deficiency increased lethal effects, indicating that the toxin can also have a protective function. Indeed, with UV irradiation the toxin was protective at low stress levels and toxic at high ones. Thus, a single TA system can exert opposing effects, a feature that may help explain the conflicting conclusions reached previously from studies with *E. coli*. The observation of opposing effects leads to the hypothesis that toxin action may play a central role in determining whether a stressful event is mild enough to be overcome by repair or so severe that a death pathway is activated.

## Results

### Bacteriostatic effects of stress are unaffected by an *ndoA* deficiency

The *B. subtilis ndoA* (toxin) gene was inactivated by homologous recombination using a plasmid-borne deletion. No growth defect was evident when mutant cells were grown in LB medium (Fig. S1), nor did the toxin deficiency affect growth inhibition caused by chloramphenicol, ciprofloxacin, kanamycin, mitomycin C, moxifloxacin, or rifampicin, as measured by minimal inhibitory concentration (MIC; Table 1). This absence of a toxin effect on antimicrobial-mediated growth inhibition allowed lethal activity to be compared under various conditions without normalizing absolute drug concentrations to MIC (such normalization is often needed to minimize contributions from factors, such as efflux and drug uptake, that could affect antimicrobial lethality through changes in intracellular drug concentration).

**Table 1:** Antimicrobial susceptibility (Minimal Inhibitory Concentration (MIC)) with *B. subtilis* strains.

Antimicrobial	MIC (µg/ml)^a^	
	BD630(wild type)	3169(*ΔndoA*)
Moxifloxacin	0.025	0.025
Kanamycin	0.4	0.4
Ciprofloxacin	0.05	0.05
Oxolinic acid	0.25	0.25
Rifampicin	0.1	0.1
Chloramphenicol	3.2	3.2
Mitomycin C	0.1	0.1

a MIC was determined in three replicate experiments; values were the same in each replicate.

### An *ndoA* deficiency decreases antimicrobial lethality

**Figure 1:**
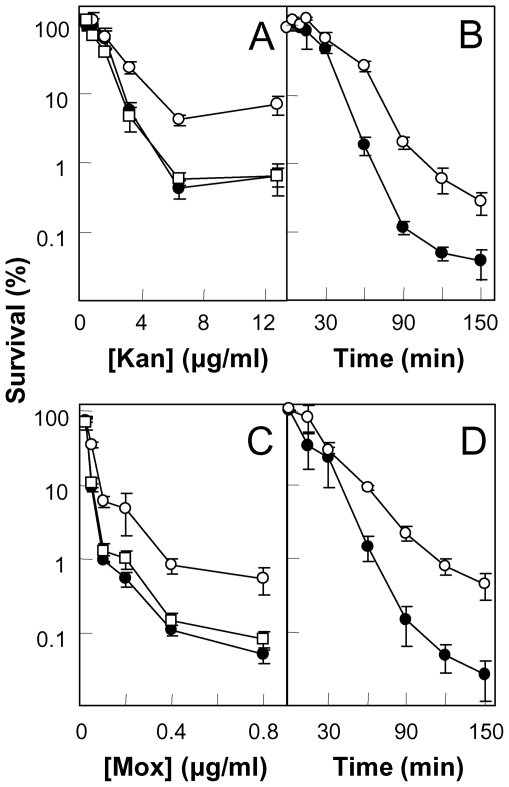
Effect of *ndoA* deficiency on bacterial survival after antimicrobial treatment. Wild-type strain (BD630, filled circles) and its *ΔndoA* mutant (3169, open circles) were treated with the indicated concentrations of kanamycin (Kan) for 1 hr (panel A), 10-times MIC (4 µg/ml) kanamycin for the indicated times (panel B), the indicated concentrations of moxifloxacin (Mox) for 2 hr (panel C), and 16-times MIC (0.4 µg/ml) moxifloxacin for the indicated times (panel D). Results with a complemented *ΔndoA* mutant strain (3322) containing a single copy of the intact *ndoAI-ndoA* operon inserted at the chromosomal *amyE* locus are shown in panels A and C (open squares). Percent survival was determined as in Methods. Error bars indicate standard deviation; similar results were obtained in replicate experiments.

To determine whether an *ndoA* deficiency is protective or detrimental during lethal stress, we first exposed cells to antimicrobial treatment. When wild-type and *ndoA*-deficient cells were treated with various concentrations of kanamycin for 1 hr (Fig. 1A) or with 4 µg/ml kanamycin (10 times MIC) for various periods of time (Fig. 1B), survival of the *ndoA* mutant was 10- to 20-fold greater than that of wild-type cells. These data indicate that in wild-type cells the NdoA toxin contributes to the lethal activity of kanamycin. Protection from kanamycin-mediated killing by the *ndoA* deficiency was eliminated when a copy of *ndoA* was integrated into the *amyE* locus of the *ndoA*-deficient strain (Fig. 1A, open squares). Thus, the protective phenotype observed was due to the absence of the *ndoA* gene product rather than to *cis*-acting effects arising from insertion of an antibiotic marker into the *ndoA* locus.

To determine whether a similar phenomenon occurs with other antimicrobials, we treated wild-type and toxin (*ndoA*)-deficient cells with various concentrations of the fluoroquinolone moxifloxacin. The *ndoA* deficiency caused survival to increase by about 10-fold (Fig. 1C). The toxin deficiency also increased survival by 10- to 20-fold when *B. subtilis* was exposed to moxifloxacin for various times at 16-times MIC (Fig. 1D). Complementation of the protection afforded by the *ndoA* deficiency to the lethal action of moxifloxacin was observed when an intact copy of *ndoA* was integrated at the chromosomal *amyE* locus (Fig. 1C). The *ndoA* deficiency also increased survival during treatment with ciprofloxacin, oxolinic acid, and mitomycin C (Fig. S2). Thus, in wild-type cells the NdoA toxin increases the lethal action of several diverse antimicrobials.

### An *ndoA* deficiency can both increase and decrease the effects of lethal stress

**Figure 2:**
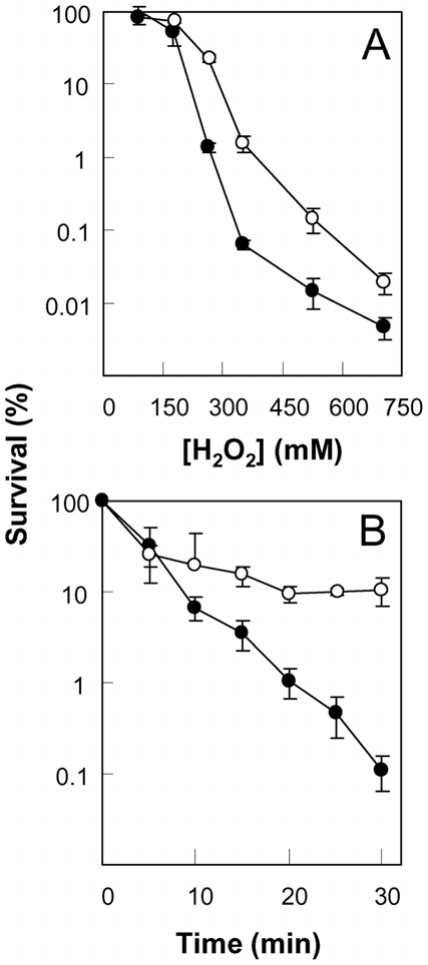
Effect of *ndoA* deficiency on bacterial survival after treatment with hydrogen peroxide. Wild-type strain (BD630, filled circles) and its *ΔndoA* mutant (3169, open circles) were treated with the indicated concentrations of hydrogen peroxide for 15 min (panel A) or for the indicated times at 250 mM (panel B). Error bars indicate standard deviation; similar results were obtained in replicate experiments.

To determine whether an *ndoA* deficiency also affects the bacterial response to commonly encountered environmental stressors, we examined effects of toxin deficiency on the lethal action of oxidative stress, UV irradiation, and temperature. When wild-type and toxin-deficient cells were compared for survival after a 15-min exposure to various concentrations of hydrogen peroxide, the *ndoA* deficiency increased survival by 5–30 fold (Fig. 2A). An even greater protective effect (up to 100-fold) was observed when cells were treated with 250 mM hydrogen peroxide for various times (Fig. 2B).

**Figure 3:**
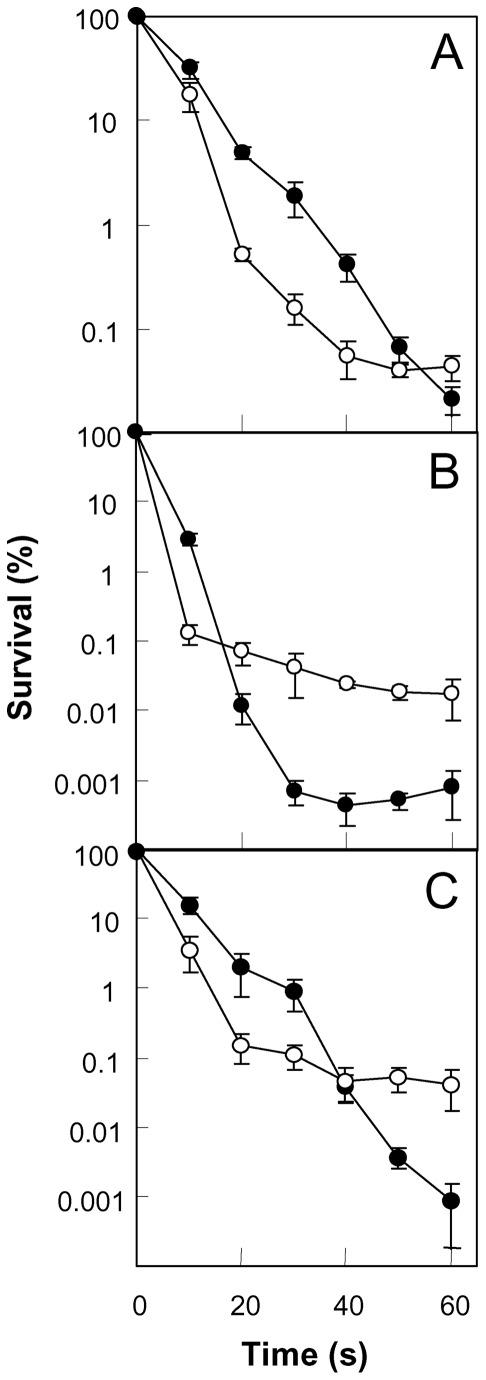
Effect of *ndoA* deficiency on bacterial survival after treatment with UV irradiation. Overnight cultures of *B. subtilis* were diluted with LB medium, 10-µl aliquots were spotted on LB agar plates, and spots were allowed to dry. The plates were exposed to UV irradiation (254 nm) at various intensities for the indicated times, and they were incubated overnight at 37°C. Wild-type strain (BD630, filled circles) and its Δ*ndoA* mutant (3169, open circles) received low (0.14 mW/cm^2^, panel A), high (0.4 mW/cm^2^, panel B), or moderate exposure (0.2 mW/cm^2^, panel C). Error bars indicate standard deviation; similar results were obtained in replicate experiments.

Exposure to UV irradiation at different levels revealed opposing effects of NdoA function. The mutant was hypersensitive to low levels of UV irradiation (0.14 mW/cm^2^, Fig. 3A). But increasing the irradiance intensity to 0.4 mW/cm^2^ showed that the mutant was less sensitive than wild-type cells at high UV exposures (Fig. 3B). At moderate irradiance intensity (0.2 mW/cm^2^), the *ndoA* mutant was 5–10 fold more sensitive than wild-type cells with short exposure times (10 to 30 sec), but the mutant became less sensitive when exposure was longer (beyond 30 sec; Fig. 3C). This cross-over from being more sensitive to less sensitive suggests that the effect of the toxin may depend on stress level, at least with some types of stress, such as UV irradiation. Interestingly, the crossover points seen in the 3 panels of Fig. 3 all occurred at about the same level of accumulated energy (e.g. 7–8 milliJoules/cm^2^, Figure S3), which indicated that a threshold in accumulated energy rather than in irradiance intensity or exposure time determines whether the NdoA toxin exhibits a protective or detrimental effect.

**Figure 4:**
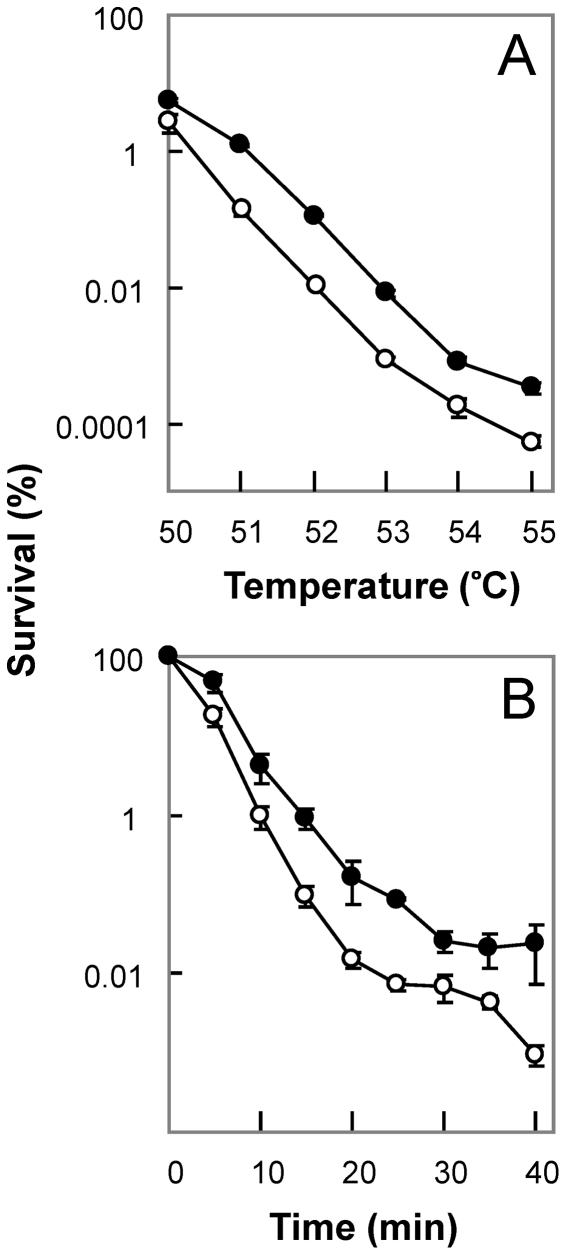
Effect of *ndoA* deficiency on bacterial survival during high-temperature treatment. Overnight cultures of wild-type *B. subtilis* strain (BD630, filled circles) and its *ΔndoA* mutant (3169, open circles) were concentrated by centrifugation, washed in saline (0.9% NaCl), resuspended and serially diluted in saline, and incubated at the indicated temperatures for 20 min (panel A) or for the indicated times at 52°C (panel B). After incubation, the cultures were diluted and applied to LB agar plates for colony determination. Error bars indicate standard deviation. Similar results were obtained in replicate experiments.

When *B. subtilis* was incubated at temperatures ranging from 50°C to 55°C for 20 min, the colony-forming capability of the *ndoA* mutant was 5- to 10-fold lower than that of the wild-type strain (Fig. 4A). A slightly greater hypersensitivity was observed when cells were incubated at 52°C for various times (Fig. 4B). No cross-over from the hypersensitive state to a protected state was observed for the mutant despite examination of several combinations of temperature and incubation time (not shown). Thus, wild-type toxin exerts only a protective effect with some types of stress, such as heat, unlike the dual action seen with UV irradiation and the detrimental effect shown for antimicrobials.

### An *ndoA* deficiency suppresses sporulation

**Figure 5:**
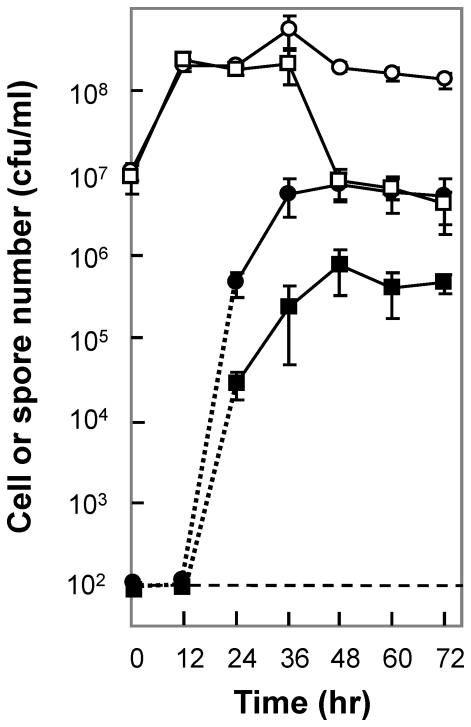
Effect of *ndoA* deficiency on bacterial sporulation and survival. *B. subtilis* wild-type strain (BD630, circles) and its *ΔndoA* mutant (3169, squares) were incubated in Difco sporulation medium. At the indicated times, aliquots were removed and incubated at 90°C for 20 min to kill vegetative cells. Total bacterial cell (before heat, open symbols) and spore numbers (after heat, filled symbols) were plotted as a function of time. Dotted lines indicate extrapolation to detection limit (100 cfu/ml, dashed line). Error bars indicate standard deviation; similar results were obtained in replicate experiments.

Since a MazF-like toxin has been implicated in myxobacterial fruiting body formation, a developmental process that leads to mother cell lysis and spore generation [23], we next examined the effect of the *ndoA* mutation on sporulation. *B. subtilis* cultures were incubated in Difco sporulation medium, and aliquots taken at various times were treated for 20 min at 90°C to kill vegetative cells. Spore number increased gradually as incubation time increased from 12 to 36 hr, with the wild-type culture forming 10–40 fold more spores than the *ndoA* mutant (Fig. 5, filled symbols). No difference in total cell number was evident during the first 36 hr of incubation (Fig. 5, open symbols). When incubation was extended to longer times, spore number stopped increasing with both wild-type and mutant cells. However, after incubation for 48 hr, the total bacterial number (CFU before heat treatment) declined (by 20–30 fold) with the *ndoA* mutant but not with the wild-type cells (Fig. 5, open squares). These data indicate that NdoA contributes not only to sporulation, but also to long-term survival under nutrient-limiting conditions.

### An *ndoA* deficiency blocks antimicrobial-mediated peroxide accumulation

**Figure 6:**
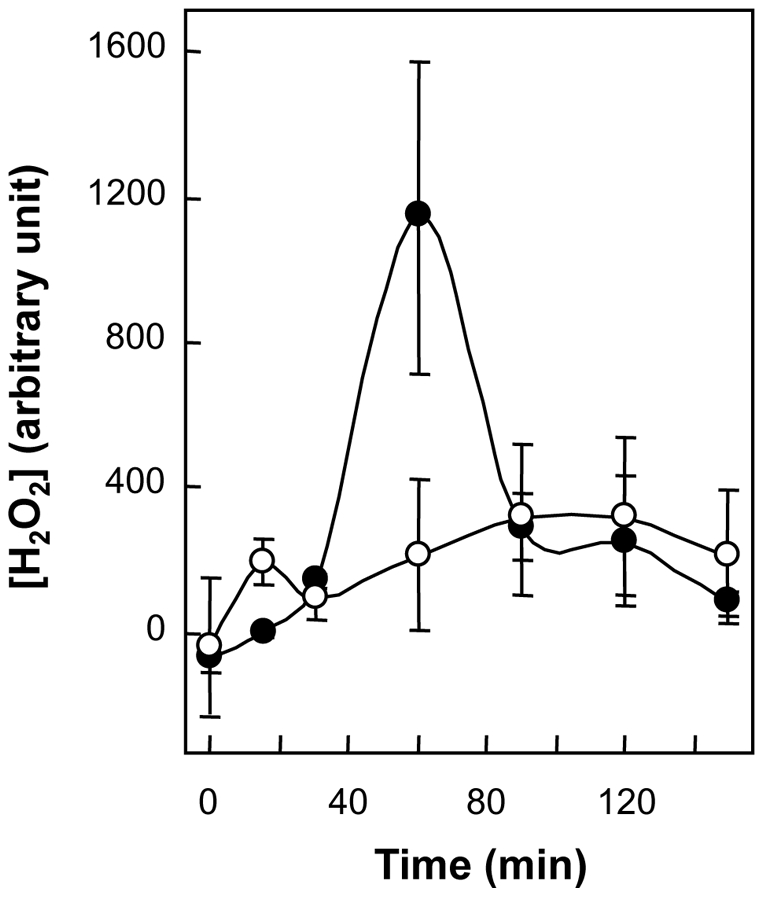
Effect of *ndoA* deficiency on peroxide accumulation following kanamycin treatment. Wild-type *B. subtilis* strain BD630 (filled circles) and its *ΔndoA* mutant 3169 (open circles) were grown overnight, diluted 50-fold in LB medium, and treated with kanamycin (4 µg/ml). At the indicated times aliquots were removed for determination of hydrogen peroxide as in Methods. Error bars indicate standard deviation; Similar results were obtained in replicate experiments.

In work described above, the presence of the NdoA toxin enhanced the lethal effects of kanamycin and moxifloxacin (Fig. 1). Since the lethal action of several antimicrobial agents is associated with production of reactive oxygen species [24], [25], [26], [27], we next measured peroxide accumulation associated with kanamycin treatment. As shown in Fig. 6, kanamycin exposure resulted in an NdoA-dependent surge of hydrogen peroxide; the absence of the toxin eliminated the surge (Fig. 6). Similar results were obtained with moxifloxacin-treated cells (not shown). Thus, the lethality-enhancing effects of the NdoA toxin correlate with peroxide accumulation.

## Discussion

The data described above show that the single type-2 toxin encoded by the *B. subtilis* genome can exert two opposing effects under stress: it protects from moderate levels of lethal stress, yet it amplifies the bactericidal action associated with high stress levels. One example was seen with UV irradiation. As UV exposure increased, an *ndoA* toxin-deficient mutant initially exhibited hypersensitivity to UV stress, and when the level of UV exposure increased beyond a threshold, the mutant became less sensitive than wild-type cells (Fig. 3). For antimicrobials and hydrogen peroxide, the enhancement of lethality by the toxin was readily observed. In contrast, a protective effect of toxin activity was observed when cells were exposed to high temperature (Fig. 4). Likewise, the toxin promoted sporulation and long-term survival under the nutrient-limiting conditions that induce sporulation (Fig. 5). Thus, the type of stress may also determine whether the toxin protects from stress or pushes the stressed cells toward death. We propose that in the case of mild stress in which damage can be repaired, the toxin favors a protective pathway. However, when stress is extreme, enhanced lethality is favored, perhaps to conserve resources for surviving members of the population.

The protective effect of TA modules at low-to-moderate levels of stress is best characterized for the MazF toxin of *E. coli*. This protein cleaves RNA at ACA sites present in most *E. coli* mRNAs. Consequently, toxin activity blocks translation. Indeed, removal of MazF-mediated cleavage sites from mRNAs encoding a variety of human, yeast, and bacterial proteins allows preferential, high-level expression of these proteins following induced, high-level expression of MazF [28]. A protective activity due to inhibition of translation is consistent with the observation that blocking protein synthesis by chloramphenicol protects *E. coli* from the lethal action of a variety of stressors such as some quinolones, kanamycin, UV irradiation, and H_2_O_2_ [29], [30], [31], [32], [33]. The *B. subtilis* NdoA toxin, which is similar to the *E. coli* MazF protein, probably acts in a similar manner when protecting cells from low levels of lethal stress.

Several lines of evidence support the argument that the deleterious action of the toxin is mediated by reactive oxygen species. In one line, kanamycin- and moxifloxacin-mediated surges of peroxide in *B. subtilis* require NdoA toxin (Fig. 6 and data not shown). In a second line, SoxR and SoxS, regulators that are responsive to superoxide concentration, were identified with *E. coli* as survival proteins whose synthesis is increased when MazF is activated by antimicrobial stress (synthesis of most other proteins under the same conditions decreases [34]). Third, addition of catalase to growth medium or over-expression of *katE* or *sodA*, which express catalase and superoxide dismutase, respectively, suppress MazF-mediated cell death triggered by extreme antimicrobial treatment [35]. Fourth, antimicrobial stress triggers production of elevated levels of carbonylated cellular proteins, which are eliminated when *mazEF* is deleted or when catalase is supplied either by exogenous addition to the growth medium or by endogenous over-expression from a plasmid [35]. Cell death probably arises from hydroxyl radical rather than peroxide, because in *E. coli* peroxide treatment fails to kill cells when hydroxyl radical accumulation is blocked (X. Wang and X. Zhao, unpublished observation). Understanding how toxins stimulate reactive oxygen species may provide new ways to manipulate the lethal activity of many antimicrobials simultaneously.

Additional responses may initiate from toxin-mediated protein truncation. When lethal stress releases toxin activity that fragments mRNA, it produces truncated proteins. Some of the truncated proteins may insert into membranes and activate the Cpx two-component envelope-stress-response system. Cpx then activates the Arc two-component system, which perturbs the respiratory chain and leads to a surge in superoxide [16], [26]. We propose that the size of this surge contributes to a live-or-die decision. Low levels of superoxide appear to be protective, because sublethal concentrations of metabolic generators of superoxide (paraquart or plumbagin) protect *E. coli* from the lethal action of bleomyin [36] and ciprofloxacin (M. Mosel, K. Drlica, and X. Zhao, unpublished observations). Moreover, a *sodAB* double mutant, which may have an elevated level of intracellular superoxide due to lack of enzymatic dismutation, also exhibits a protective rather than a detrimental effect [27]. Such protective effects may be triggered by low-to-moderate levels of superoxide being able to activate s*oxRS* and derepress *marR*, both of which can activate expression of the *mar* (multiple antibiotic resistance pump) system [37], [38], [39]. Activated SoxS may also trigger other protective systems (e.g. *nfo* (M. Mosel, K. Drlica, and X. Zhao, unpublished observations)) for repair of stress-mediated damage. In contrast, high levels of superoxide lead to production of peroxide and then toxic hydroxyl radical, as argued by Collins [25], [26]. Hydroxyl radical accumulation then leads to cell death. Thus, the level of stress detected by TA systems would stimulate either a protective (*soxS-marR*) or hyperlethal (ROS) response.

TA modules also appear to participate in complex developmental responses to stress. For example, a MazF-like toxin contributes to the death of mother cells of *Myxococcus xanthus* during fruiting body (spore) formation [23]. In the present work, we found that the absence of toxin led to a decrease in the ability of *B. subtilis* to sporulate and to survive long-term incubation (Fig. 5). In contrast, the toxin deficiency slightly increased (∼3 fold) the ability of *B. subtlis* to develop competence, as determined by bacterial chromosome transformation (not shown). The toxin knockout mutant of *B. subtilis* also exhibited a 10- to 40-fold reduction in the frequency at which ciprofloxacin-resistant and rifampicin-resistant mutants were selected on agar when the drugs were present at moderate concentrations (2- to 6-times MIC; not shown). Thus, a single toxin-antitoxin module can affect many aspects of bacterial physiology.

In addition to protein-protein (type-2) TA modules, TA pairs comprised of RNA components (type-1 TA) have also been discovered in bacteria [40], [41]. *B. subtilis* contains one such module [42]. In type-1 TA systems, the antitoxin is a small RNA, rather than a labile protein, that forms complementary base-pairs with the toxin mRNA to suppress toxin gene translation [40], [41]. Type-1 toxins are usually smaller and more hydrophobic than type-2 toxins. The biochemical activity of type-1 toxins is unknown, although a similarity to phage holing proteins and peptide antimicrobials has been noted [40]. Whether type-1 and type-2 TAs respond to stress in a similar way remains to be established experimentally.

### Conclusions

The *B. subtilis* NdoAI-NdoA toxin-antitoxin module can either enhance or diminish the effects of lethal stress depending on the type and level of stress. The toxin increases the lethal action of antimicrobials, such as kanamycin and moxifloxacin, while it decreases killing by heat and nutrient starvation. With UV irradiation, the toxin reduces killing at low doses but increases it at high doses.

Having two effects fits data reported for the MazEF TA module of *E. coli*. Thus, bacteria appear to have pathways that allow cells to recover from mild stress but undergo ROS-mediated death when stress is severe.

## Materials and Methods

### Chemicals and reagents

Rifampicin, erythromycin, oxolinic acid, mitomycin C, horseradish peroxidase, 10-acetyl-3,7-dihydroxyphenoxazine (Amplex Red), and hydrogen peroxide were obtained from Sigma Chemical Co. (St. Louis, MO). Ciprofloxacin and moxifloxacin were provided by Bayer AG (West haven, CT). Kanamycin was purchased from Fisher Scientific (Fairlawn, NJ), chloramphenicol was from EMD Chemicals, Inc. (Gibbstown, NJ), and ampicillin was a product of Roche Diagnostics (Indianapolis, IN).

### Bacterial strains, growth conditions, and susceptibility assays

Strains of *B. subtilis* used in the work were derivatives of strain BD630 obtained from Dr. D. Dubnau, PHRI. Strain 3169 was deleted for *ndoA* and was constructed as described below. Strain 3322 was a derivative of strain 3169 in which a copy of native *ndoA* was integrated into the *amyE* locus as described below. *B. subtilis* was grown at 37°C in LB broth with shaking at 250 rpm or on LB agar [43] unless specified otherwise.

Plasmid pBS-NdoA-Ery, which contains an erythromycin (*ery*)-resistance cassette bounded by homologous regions upstream and downstream of *ndoA*, was used to inactivate the toxin gene (*ndoA*) by homologous recombination. The plasmid was obtained from Ciaran Condon [21] and was introduced into wild-type *B. subtilis* strain BD630 by bacterial transformation to generate the *ndoA* mutant (strain 3169). The deficiency of the targeted *ndoA* gene in the mutant was confirmed by PCR-mediated amplification of nucleotides between the upstream gene *alr* and the *ery* cassette, using primers Alr-F (5′-CAT GGA TGA GAT TGC AGG AAG-3′) and Ery-R (5′-GAG GTG TAG CAT GTC TCA TTG-3′) followed by gel electrophoresis analysis of product size and by nucleotide sequence determination as in [21].

Growth inhibition (MIC) was determined by broth dilution using a series of tubes, each containing about 10^5^ bacteria in 1 ml of LB liquid medium supplemented with antimicrobials at various concentrations that differed by 2-fold increments. The lowest drug concentration that prevented visible growth after overnight incubation was taken as MIC.

Rapid killing of bacterial cells was measured with cultures that had been grown overnight (about 17 hrs) and then diluted by 50-fold before treatment with various stressors for various times. After treatment, cells were diluted with LB medium and applied to LB agar plates lacking stressor. Colonies were counted after overnight incubation at 37°C. Untreated cells, taken at the time stress was applied, served as a time-zero control; survival was calculated relative to CFU at time zero.

### Complementation of *ndoA* deficiency-mediated protection from antimicrobial lethality

The *ndoAI-ndoA* operon containing a 484-bp upstream promoter region was amplified by PCR and cloned into plasmid pDR66 [44] between the *Bam HI* and *Eco RV* restriction sites. This strategy allows removal of the P_spac_ promoter from pDR66 and placement of a native copy of the *ndoAI-ndoA* operon between two DNA segments of the *amyE* locus. A single copy of *ndoAI-ndoA* was then integrated into the chromosomal *amyE* locus via homologous recombination. Confirmation of correct integration of *ndoA* into the *amyE* locus of the *ndoA* mutant (strain 3169) was achieved by PCR (using one primer derived from the pDR66 vector and another primer from the end of *ndoA*) and nucleotide sequence determination. The newly constructed strain (3322), along with the wild-type (BD630) and the *ndoA* mutant (strain 3169), were examined for susceptibility to the lethal action of kanamycin and moxifloxicin.

### UV irradiation

Overnight cultures were diluted by 10^1^ to 10^6^-fold with fresh LB medium, and 3 aliquots of 10 µl for each dilution were spotted on LB agar plates. The plates were exposed in the dark to a UV (254 nm) lamp (model G18T8, General Electric Co, Cleveland, Ohio) at irradiance intensity of 0.14, 0.20, or 0.40 mW/cm^2^ for various times (10 to 60 sec). The plates were then incubated overnight at 37°C in the dark. Survival was determined relative to CFU measured without UV treatment.

### Sporulation assay

A single *B. subtilis* colony grown for 24-hr was inoculated into Difco sporulation medium [45] and incubated at 37°C with shaking at 300 rpm for various times. Aliquots were removed periodically, and half of each sample was incubated at 90°C for 20 min to kill vegetative cells. Samples were diluted and plated on LB agar for determination of spore number. The other half of each sample was diluted and plated without heating for determination of total bacterial counts. Sporulation frequency was taken as the ratio of CFU after heat treatment to CFU before heat treatment.

### Hydrogen peroxide assay

Bacteria were treated with 4 µg/ml (10 MIC) kanamycin for various times after which cells were harvested by centrifugation at 10,000× g for 5 min. Cells were resuspended in 0.9% NaCl, and centrifugation was repeated. Hydrogen peroxide concentration was measured as described [46] by horseradish peroxidase- (4 units) and H_2_O_2_-mediated oxidation of 50 µM 10-acetyl-3,7-dihydroxyphenoxazine (Amplex Red) to the highly fluorescent compound resorufin [47]. Fluorescence was measured using a Spectramax M2 microplate reader (Molecular Devices, Sunnyvale, CA) at 587 nm with excitation at 563 nm.

## Supporting Information

Figure S1 **Effect of** ***ndoA*** **deficiency on bacterial growth.** Wild-type strain (BD630, filled circles) and its *ΔndoA* mutant (3169, open circles) were grown as single colonies on LB agar by overnight incubation at 37°C. A single colony was inoculated into 5 ml of LB broth and incubated at 37°C. Bacterial growth was monitored as turbidity increase at the indicated times. Error bars indicate standard deviation.
(TIF)

Figure S2 **Effect of** ***ndoA*** **deficiency on bacterial survival after treatment with ciprofloxacin, oxolinic acid, and mitomycin C.** Wild-type strain (BD630, filled circles) and its *ΔndoA* mutant (3169, open circles) were treated with the indicated concentrations of ciprofloxacin for 120 min (panel A), oxolinic acid for 180 min (panel B), or mitomycin C for 30 min (panel C). Error bars indicate standard deviation; similar results were obtained in replicate experiments.
(TIF)

Figure S3 **Effect of accumulated UV irradiation energy on differential killing of wildtype and a** ***ndoA*** **deficient mutant.** Percent survival of wild-type strain (BD630, filled symbols) and its Δ*ndoA* mutant (3169, open symbols) received low (0.14 mW/cm^2^, diamonds), high (0.4 mW/cm^2^, circles), or moderate (0.2 mW/cm^2^, triangles) UV irradiation intensity, as displayed in Figures 3A–3C, is plotted as a function of accumulated energy (milliJoule/cm2) in a single panel to show that bacterial killing correlates with accumulated radiation energy and that the cross-over between the wild-type and the mutant data occurs at the same accumulated energy level.
(TIF)
