# Supplementary figures and images for: A Toxin-Antitoxin Module in Bacillus subtilis Can Both Mitigate and Amplify Effects of Lethal Stress
Source: PLoS One. 2011 Aug 29;6(8):e23909. doi: 10.1371/journal.pone.0023909 (PMC3163665; doi:10.1371/journal.pone.0023909)

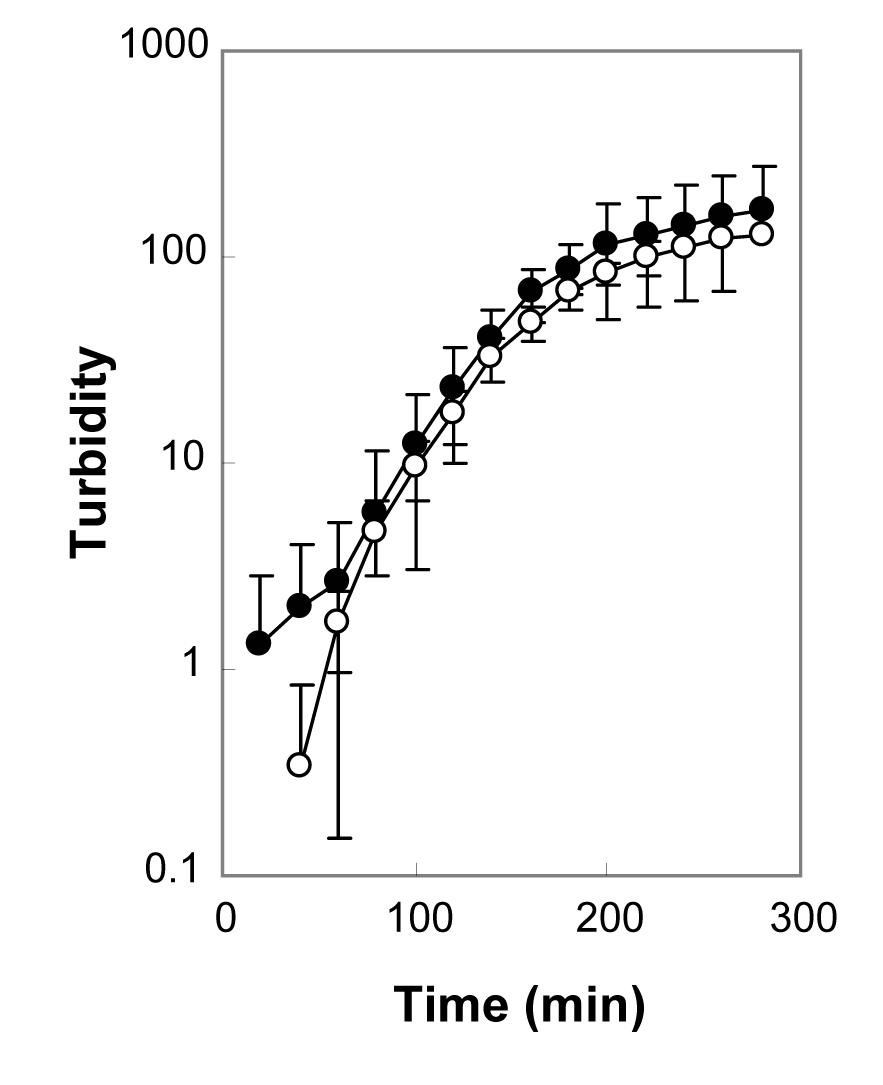

Supplement: Figure S1 — Effect of ndoA deficiency on bacterial growth. Wild-type strain (BD630, filled circles) and its ΔndoA mutant (3169, open circles) were grown as single colonies on LB agar by overnight incubation at 37°C. A single colony was inoculated into 5 ml of LB broth and incubated at 37°C. Bacterial growth was monitored as turbidity increase at the indicated times. Error bars indicate standard deviation. (TIF) [file pone.0023909.s001.tif]

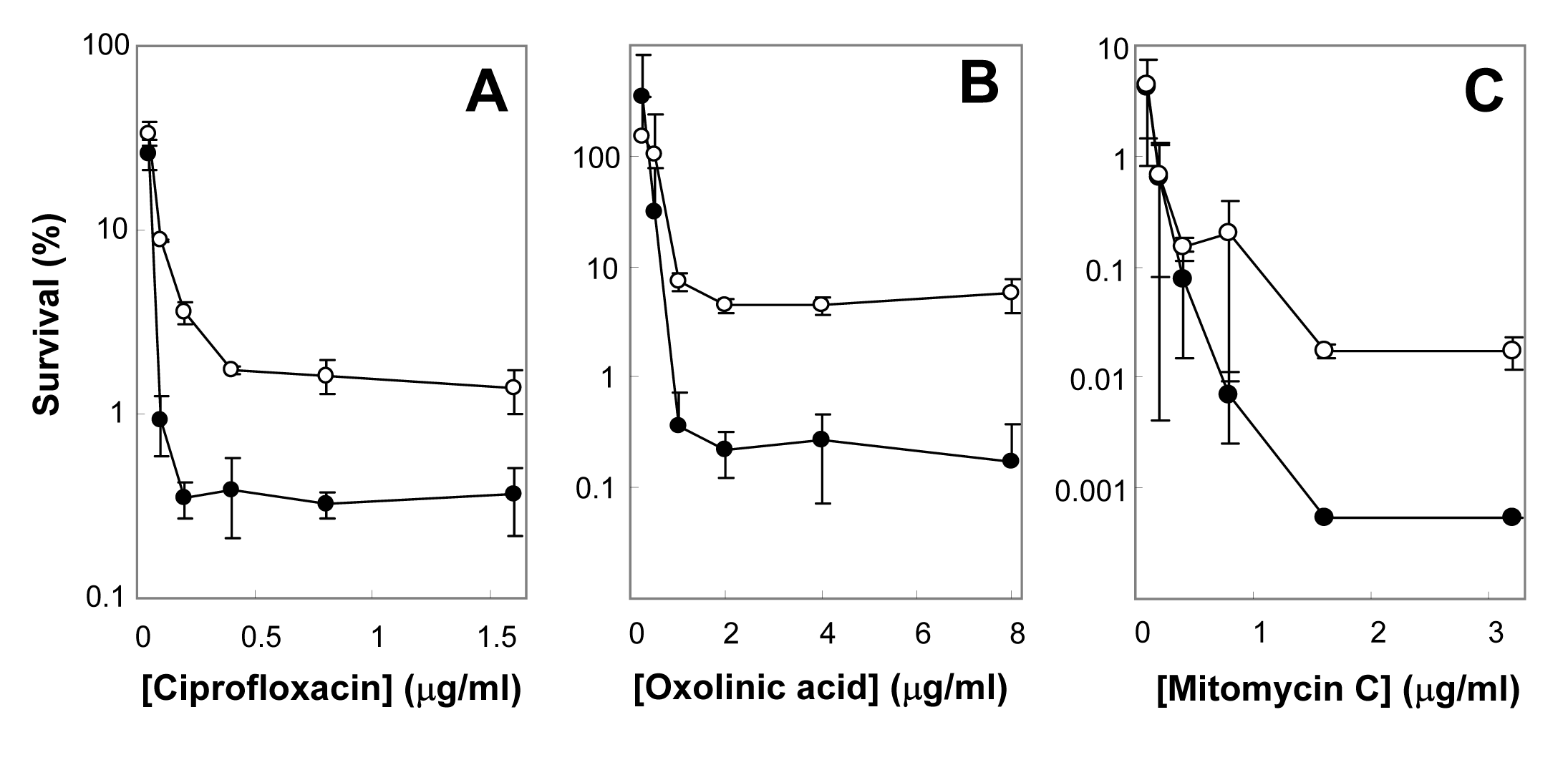

Supplement: Figure S2 — Effect of ndoA deficiency on bacterial survival after treatment with ciprofloxacin, oxolinic acid, and mitomycin C. Wild-type strain (BD630, filled circles) and its ΔndoA mutant (3169, open circles) were treated with the indicated concentrations of ciprofloxacin for 120 min (panel A), oxolinic acid for 180 min (panel B), or mitomycin C for 30 min (panel C). Error bars indicate standard deviation; similar results were obtained in replicate experiments. (TIF) [file pone.0023909.s002.tif]

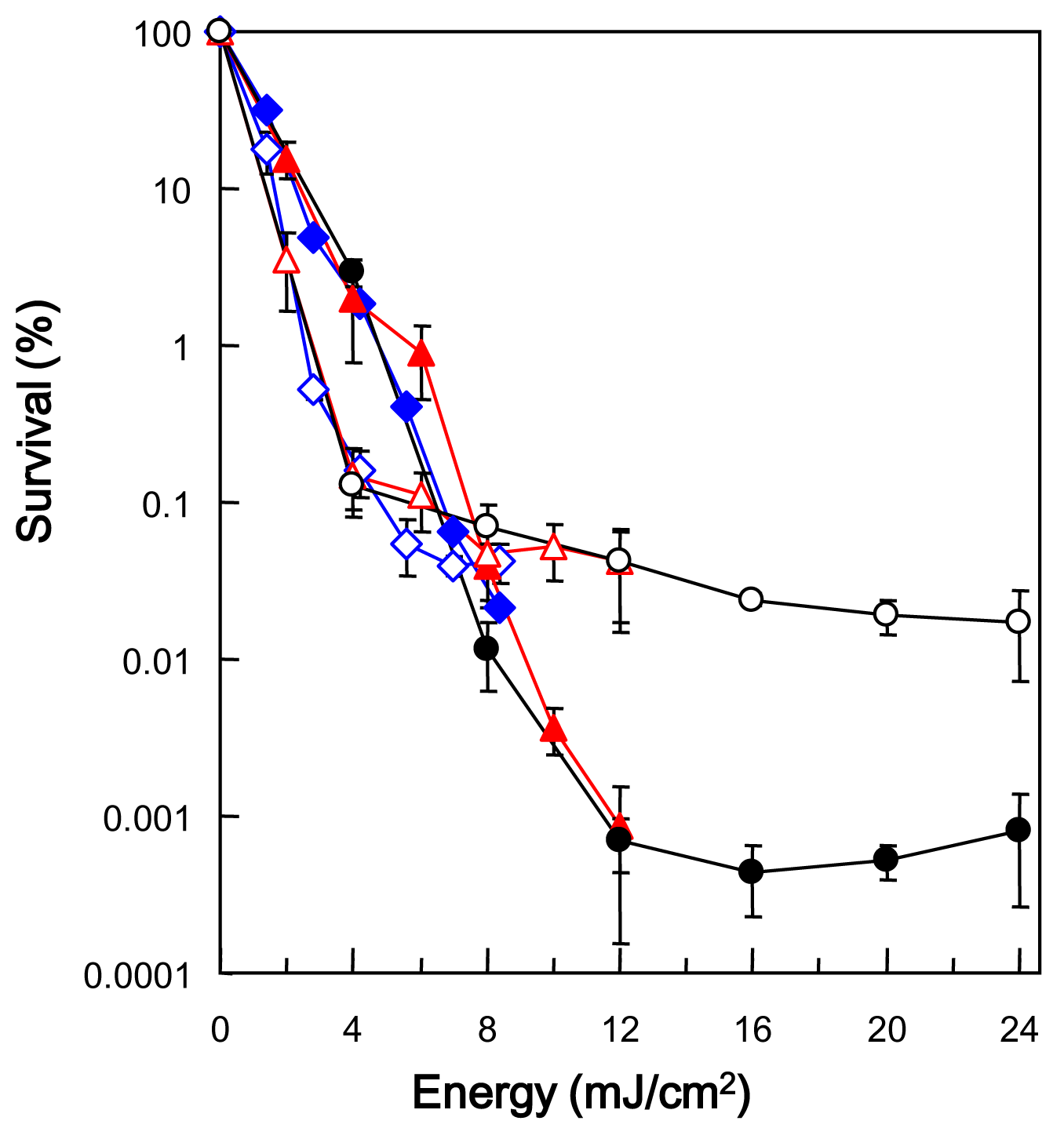

Supplement: Figure S3 — Effect of accumulated UV irradiation energy on differential killing of wildtype and a ndoA deficient mutant. Percent survival of wild-type strain (BD630, filled symbols) and its ΔndoA mutant (3169, open symbols) received low (0.14 mW/cm2, diamonds), high (0.4 mW/cm2, circles), or moderate (0.2 mW/cm2, triangles) UV irradiation intensity, as displayed in Figures 3A–3C, is plotted as a function of accumulated energy (milliJoule/cm2) in a single panel to show that bacterial killing correlates with accumulated radiation energy and that the cross-over between the wild-type and the mutant data occurs at the same accumulated energy level. (TIF) [file pone.0023909.s003.tif]
